# Psychological monitoring in isolated, confined, and extreme environments: promise and challenges of ecological momentary assessment

**DOI:** 10.3389/fphys.2026.1695483

**Published:** 2026-08-13

**Authors:** Clara Richard

**Affiliations:** Life Sciences, NeurAstra, Annecy, France

**Keywords:** ecological momentary assessment, extreme environments, human factors, polar psychology, psychological monitoring, spaceflight psychology

## Abstract

Personnel in Isolated, Confined, and Extreme (ICE) environments face unique psychological stressors that can compromise performance, safety, and mission success. Traditional psychological monitoring approaches, predominantly retrospective questionnaires and clinical interviews, may inadequately capture the dynamic nature of psychological adaptation in these demanding contexts. This mini-review examines the potential role of Ecological Momentary Assessment (EMA) as part of a comprehensive approach to psychological monitoring in ICE environments. We review current monitoring practices across spaceflight, polar, submarine, and remote field operations, and evaluate emerging technologies including EMA, wearable sensors, and digital biomarkers. While EMA offers theoretical advantages through real-time data collection and reduced recall bias, implementation faces substantial obstacles including technological constraints, participant burden, cultural considerations, and validation challenges. Limited empirical evidence suggests that hybrid approaches combining multiple assessment modalities may be most promising for operational implementation. We propose a framework for developing next-generation psychological monitoring systems that balance scientific rigor with operational feasibility, emphasizing environment-specific validation and the inherent sample-size constraints of ICE settings.

## Introduction

The expansion of human activity into increasingly challenging environments—from long-duration spaceflight missions to Antarctic research stations, submarine operations, and remote expeditionary contexts—has highlighted critical gaps in our ability to monitor and support psychological well-being under extreme conditions (Sandal et al., 2018; Palinkas, 2003).

These Isolated, Confined, and Extreme (ICE) environments impose unique psychological stressors including prolonged isolation, spatial confinement, environmental hazards, operational pressures, and interpersonal tensions arising from sustained close-quarters living and working (Sandal et al., 1995). Current psychological monitoring practices rely heavily on pre-deployment screening, periodic clinical assessments, and post-mission debriefing (Kanas et al., 2009). While providing valuable baseline data, these approaches may miss critical psychological fluctuations during operations when intervention opportunities are most important yet constrained.

The need for responsive psychological monitoring has intensified as missions become longer and more remote. Future Mars missions are expected to involve total mission durations on the order of 2–3 years, with surface stays of approximately 500–900 days depending on mission architecture (Drake, 2009), and one-way Earth–Mars communication delays of 4–24 minutes that preclude real-time clinical consultation (Landon et al., 2018). These constraints make traditional Earth-based real-time psychological support impossible and necessitate substantially greater autonomous monitoring capabilities than are required for current low-Earth-orbit operations.

Literature for this mini-review was identified through targeted searches of PubMed and Google Scholar between May 2025 and May 2026, spanning the original drafting and revision periods. Search terms combined variants of ‘ecological momentary assessment,’ or ‘experience sampling,’ with domain terms including ‘spaceflight,’ ‘astronaut,’ ‘Antarctic,’ ‘winter-over,’ ‘submarine,’ ‘isolated confined extreme,’ and ‘ICE environments.’ Additional references were identified through backward citation tracing from key review articles in the field (notably Kanas et al., 2009; Palinkas and Suedfeld, 2008; Sandal et al., 2018) and from foundational methodological papers on ecological momentary assessment (Shiffman et al., 2008). Given the mini-review format, the search was not intended to be exhaustive but rather to identify representative empirical studies, methodological papers, and reviews spanning the four operational contexts under discussion.

## Current monitoring practices and limitations

### Spaceflight operations

Current International Space Station (ISS) operations employ weekly Private Psychological Conferences with ground-based clinicians and periodic standardized questionnaires, supplemented by behavioral observation and operational performance data (Kanas et al., 2009; Strangman et al., 2014). Although ISS operations have been broadly successful in supporting crew psychological well-being for more than two decades, scheduled assessments by design sample crew states only at fixed intervals, and mood or behavioral changes that emerge and resolve between contacts can therefore go undetected—a limitation that becomes more consequential on missions exceeding six months.

Mars missions present qualitatively different challenges that current ISS-style practices cannot fully accommodate. The 4–24 minute one-way communication delay precludes real-time consultation; resupply and crew evacuation are not feasible; and crews must operate with substantial clinical autonomy from Earth for surface stays of up to ~900 days (Drake, 2009; Landon et al., 2018). These conditions make on-board, autonomous psychological monitoring not merely useful but operationally necessary. Engineering targets, such as reliability, power, mass, and durability, should be derived from systems engineering analysis tailored to the specific mission, not asserted as universal thresholds.

### Polar and submarine operations

Antarctic research programs face psychological challenges throughout the year, particularly during 6–9 month winter-over periods. These periods are characterized by complete absence of transport for resupply, medical evacuation, or rotation of personnel, and by extreme photoperiod conditions—months of continuous darkness at high-latitude inland stations such as Concordia and Amundsen–Scott, and continuous daylight in mid-summer (Palinkas and Suedfeld, 2008). Current monitoring practice typically combines pre-deployment psychological screening, periodic structured self-report questionnaires, and informal observation by station leaders or medical officers, with little or no real-time component. Analyses of multi-year Antarctic data indicate that a substantial minority of winter-over personnel experience clinically significant mood disturbances, and that traditional monthly assessments can miss transient acute episodes that resolve between evaluations (Steel, 2005).

Submarine operations present a distinct profile. Routine in-mission psychological assessment is rare; current practice is generally limited to pre-deployment psychological screening, command-level observation by medical officers and the chain of command, and post-patrol debriefs (Chabal et al., 2018; Härmä et al., 2008). Operational tempo, watchstanding schedules, and security restrictions on radiofrequency emissions and outbound communications—particularly during patrols by ballistic missile submarines—leave little practical room for repeated structured assessment, and largely preclude transmission of psychological data to shore during a patrol. Where in-mission monitoring is contemplated, candidate equipment for psychological data collection (e.g., wearable sensors, tablet-based self-report tools, and small data-logging devices) must operate within the enclosed pressure-hull environment, withstand electromagnetic interference from sonar and other onboard systems, and tolerate long patrol durations (60+ days) during which devices cannot easily be serviced or replaced. Recent studies report that a substantial proportion of nuclear submarine crews experience significant sleep disturbances during 60+ day patrols (Chabal et al., 2018), suggesting that current monitoring practice does not fully capture or address the psychological burden of these operations.

Across all three contexts—spaceflight, Antarctic, and submarine—any candidate monitoring system must satisfy a common set of environment-specific requirements: low operational burden on crew; robustness under the relevant physical, electromagnetic, and operational conditions; compatibility with the available power, mass, volume, and communications budgets; and validated psychometric performance under stressors that closely resemble the deployment environment.

### Cultural and individual factors

Research indicates significant cultural differences in psychological adaptation patterns, with crew members from collectivistic cultures showing different help-seeking behaviors compared to individualistic cultures (Sandal et al., 2018). Individual differences in personality traits such as psychological hardiness also shape responses to prolonged operational stress and have been proposed as targets for both selection and leader-driven support (Bartone, 2006). These differences critically impact monitoring system design and interpretation.

## Ecological momentary assessment: evidence and challenges

### What is ecological momentary assessment?

Ecological Momentary Assessment (EMA) refers to a family of methods that repeatedly sample participants’ current states, behaviors, and contexts in real time within their natural environment, typically via brief prompts delivered on a portable device several times per day (Shiffman et al., 2008). EMA differs from the questionnaires and clinical interviews currently dominant in ICE settings in three respects. First, items address the present moment rather than the past week or month, reducing recall bias. Second, items are delivered repeatedly within a day or week, capturing within-person fluctuations rather than averaged or retrospectively summarized states. Third, prompts are delivered during ordinary activity rather than during a clinical contact, providing ecologically valid samples of mood, fatigue, workload, and other states as they occur in the operational context. EMA can be combined with passive sensing (e.g., wearable physiological monitoring) to triangulate self-report against objective indicators.

### Theoretical advantages and limited evidence

EMA offers several potential advantages: reduced recall bias relative to retrospective questionnaires, greater temporal precision for detecting within-person changes in mood and other states, ecological validity within operational contexts, and reduced social desirability bias through brief, automated assessments (Shiffman et al., 2008).

However, empirical evidence in ICE settings remains limited. The Mars-500 simulation demonstrated that repeated structured neurobehavioral assessments are feasible over 17-month confinement, with substantial individual variability between participants (Basner et al., 2013). Analog work at the Flashline Mars Arctic Research Station similarly demonstrated the feasibility of repeated stress, coping, and mood assessments over a 4-month simulated Mars mission, while highlighting individual and gender differences in adaptation that are relevant for monitoring system design (Bishop et al., 2010).

Reviews of polar and submarine operations describe the mood, sleep, and fatigue challenges that motivate more frequent, real-time monitoring (Chabal et al., 2018; Palinkas and Suedfeld, 2008), though direct EMA validation studies in these populations remain scarce.

### Implementation challenges

Technological constraints: The technological barriers to EMA implementation are not uniform across ICE settings; they depend on the specific physical and operational profile of each environment, and a system designed for one context will rarely transfer unmodified to another. Wearable sensors and tablet- or smartphone-based prompting devices are the most commonly contemplated platforms. In long-duration spaceflight aboard pressurized vehicles and habitats, ambient temperature and humidity are tightly controlled, so device tolerance to thermal extremes is rarely the binding constraint; the principal limits are mass and volume budgets, electrical power, on-board data storage and processing, and downlink bandwidth. For Mars missions specifically, the 4–24 minute one-way communication delay (Landon et al., 2018) precludes real-time, two-way data exchange with Earth and requires that processing and decision support be performed on-board. Submarine environments are similarly climate-controlled, but operational security and stealth requirements place strong restrictions on radiofrequency emissions and may prohibit any data transmission for the duration of a patrol; equipment must therefore log data locally for download at port. Antarctic stations have controlled interior environments but limited and expensive satellite bandwidth that is typically priced per megabyte and capped during winter; field excursions outside the station expose devices to cold well below the −10 °C lower bound at which most consumer-grade wearables fail. Remote expeditionary contexts (mountain, desert, and high-latitude field operations) face the most severe combination of conditions: temperature extremes commonly cited in the operational range −40 °C to +60 °C, broad humidity exposure (0–100%), limited power for recharging, and expensive or intermittent satellite communication.

Operational integration: Crew members in long-duration missions have limited discretionary time, requiring that monitoring tools achieve seamless integration with operational routines. Even brief interruptions may be unacceptable during critical operations.

Cultural considerations: Crew members from high-context cultures may prefer indirect communication about psychological symptoms, requiring culturally adapted protocols. Multinational crews necessitate language validation across cultures and psychological concepts that may not translate directly.

## Integration with emerging technologies

Wearable sensors providing continuous physiological monitoring complement subjective EMA reports but face accuracy degradation in extreme conditions. Digital biomarkers from communication patterns, physical activity, and device usage offer passive monitoring potential. Smartphone- and wearable-based digital phenotyping is increasingly used for behavioral and mental health monitoring in non-ICE contexts (Onnela and Rauch, 2016; Torous et al., 2017), including actigraphy-derived prediction of mood states and symptom change (Jacobson et al., 2019). Such approaches also raise privacy concerns requiring technical solutions like differential privacy.

Translating wearable and digital-biomarker data into actionable monitoring signal increasingly relies on machine learning. In ICE contexts, however, three constraints limit current AI approaches. First, training datasets are small relative to the standards of modern machine learning: cumulative samples across the full body of ICE research number in the hundreds rather than the thousands or millions that have driven progress in non-ICE digital phenotyping. Second, safety-critical applications require models whose outputs can be inspected and explained to clinicians and crew, which limits the use of complex ‘black-box’ architectures that often perform best in non-ICE benchmarks. Third, training data drawn predominantly from Western, English-speaking populations may not generalize to multinational crews. These limitations argue for human-in-the-loop systems in which algorithmic indicators support, rather than replace, clinical judgment.

## Proposed monitoring framework

Based on current evidence, we propose a tiered approach balancing scientific rigor with operational feasibility:

Tier 1 (Baseline): Pre-deployment screening with individual risk profiling and culturally-adapted protocols.

Tier 2 (Routine): Weekly structured assessments, passive physiological monitoring, and digital biomarker collection optimized for diverse cultural backgrounds. Passive physiological monitoring here refers to continuous, low-burden data streams requiring no active input from the user once the device is worn—typically resting heart rate, heart rate variability, sleep duration and architecture inferred from movement and heart-rate signals, and activity counts from a wrist-worn device. Digital biomarkers may include voice features extracted during routine communications and aggregate device-use patterns, with feature extraction performed locally on the device to address privacy concerns.

Tier 3 (Intensive): Brief daily assessments during high-risk periods, event-triggered protocols following stressful incidents, and enhanced monitoring during critical operations. For example, events such as a near-miss EVA, a major equipment failure, or notification of adverse personal news would trigger a brief stress and mood check at predefined intervals (e.g., 12 and 48 hours post-event); triggers may be entered manually by the crew member, by the on-site medical officer, or generated automatically from system telemetry.

Tier 4 (Crisis): Immediate assessment protocols, automated alerts from multiple indicators, and remote consultation when communication permits. Indicators contributing to alerts would typically include sustained reductions in sleep duration or heart rate variability relative to personal baseline, marked changes in communication or activity patterns, and self-report scores meeting pre-defined cut-offs. Alerts route first to the on-site medical officer or designated crew lead, with onward consultation to flight surgeons or shore-based clinicians as bandwidth, communication delay, and operational security permit.

Across all tiers, the framework is intended to operate primarily on locally stored, low-bandwidth data, with transmission reserved for summary indicators and crisis-level alerts — accommodating the bandwidth, communication-delay, and operational-security constraints described above.

## Validation requirements and research priorities

Robust environment-specific validation conventionally requires sample sizes around n=200 per environment for confirmatory factor analysis and measurement invariance testing (Kline, 2016), though appropriate sizes vary with model complexity and data characteristics (Wolf et al., 2013). Cultural validation requires multi-group analyses across multiple cultural groups using multiple-group structural equation modeling (Fischer and Karl, 2019).

These targets, however, are unattainable in some ICE environments simply as a matter of population size. The total population of long-duration spaceflight crew across the entire history of the ISS program is in the low hundreds, and access to operational data is further constrained by mission schedules, crew consent, and proprietary considerations. Sample sizes in submarine and Antarctic winter-over populations are larger but still modest relative to conventional psychometric targets, particularly when stratified by mission phase, role, and cultural background.

We therefore propose a complementary, multi-layered validation strategy for sample-limited contexts. First, primary psychometric validation should be conducted in high-fidelity analogs (e.g., HERA, HI-SEAS, Concordia, Halley, NEEMO, submarine patrols), where larger and more controlled samples can be aggregated through pre-registered consortia using harmonized measurement protocols. Second, hierarchical and Bayesian models with informative priors derived from analog data should be used to extract maximal information from small operational samples, treating analog evidence as prior rather than discarding it. Third, single-case experimental designs and within-person time-series methods should be used to evaluate sensitivity to change at the individual level, complementing group-level validation and aligning naturally with the within-person inferential goals of operational monitoring. Fourth, measurement invariance between analog and operational contexts should be explicitly tested rather than assumed, to determine which findings transfer and which do not.

## Future directions and implementation timeline

A systematic 10-year development approach should prioritize: (1) Years 1–3: Technology development, controlled validation studies, and cultural adaptation protocols; (2) Years 4–6: Pilot implementations in operational environments and comparative effectiveness research; (3) Years 7–10: Scaled deployment with continuous improvement and predictive analytics development.

Key development areas include predictive algorithms for early warning sign identification, personalized interventions adapting to individual profiles and cultural backgrounds, and full integration with mission planning.

## Discussion

Psychological monitoring in ICE environments represents a critical capability for ensuring mission success and personnel safety. While EMA offers promising theoretical advantages for real-time psychological assessment, the available empirical base remains limited and implementation faces substantial practical obstacles, motivating both targeted validation work and integration with complementary monitoring modalities.

Current evidence, though limited by small sample sizes and short study durations, suggests that no single monitoring approach meets all ICE requirements. Integrated systems combining multiple assessment modalities—traditional clinical approaches, brief frequent assessments, physiological monitoring, and digital biomarkers—may provide optimal capabilities while balancing scientific rigor with operational feasibility.

Within such hybrid systems, EMA plays a specific and complementary role. EMA is the principal mechanism available for capturing within-person fluctuations in mood, fatigue, workload, and stress at temporal resolutions that pre-deployment screening, periodic clinical conferences, and post-mission debriefs cannot provide. In the proposed framework, EMA is the active component of routine (Tier 2) and intensive (Tier 3) monitoring, triangulated with passive physiological and digital-biomarker streams to mitigate self-report limitations such as social desirability and reporting fatigue. Its main contribution is therefore not replacement of existing clinical practice but extension of monitoring into the operational moments — between scheduled contacts, during high-workload phases, and following critical events — where adaptation is most dynamic and intervention windows are narrowest. Realizing this role in ICE settings will require demonstrating, beyond feasibility alone, that brief repeated self-reports yield actionable signal under operational stressors and acceptable participant burden.

Priority should be given to environment-specific validation studies with adequate sample sizes, comparative effectiveness research using rigorous experimental designs, and analyses of operational and economic feasibility. Achieving these priorities under the inherent sample-size and resource constraints of ICE contexts will require deliberate methodological choices. (a) Cross-program data-sharing consortia should pool de-identified data across analogs and operational missions under harmonized protocols, allowing cumulative meta-analytic evidence to grow despite individually small studies. (b) Pre-registration and registered reports should become standard practice to maximize the inferential value of each data point and to mitigate the publication and analytic flexibility problems that disproportionately affect small-sample fields. (c) Bayesian and hierarchical analytic frameworks should be used to transfer information from analog to operational settings rather than treating each new mission as analytically independent. (d) Validation work should be embedded directly within existing missions and analog campaigns rather than run as standalone research, both reducing marginal cost and improving ecological validity. (e) A small core set of measures should be standardized across programs so that data collected for operational reasons can also serve a validation function. Cultural considerations must be systematically addressed through multinational validation studies and adapted protocols.

The frameworks and technologies developed for extreme environments may ultimately benefit psychological monitoring in less extreme contexts, contributing to broader advances in digital mental health. As missions become longer, more remote, and more challenging, investment in comprehensive psychological monitoring capabilities represents both a scientific imperative and an operational necessity for human exploration and safety in extreme environments.
